# The Interferon Response Dampens the Usutu Virus Infection-Associated Increase in Glycolysis

**DOI:** 10.3389/fcimb.2022.823181

**Published:** 2022-02-04

**Authors:** Maria Elisabeth Wald, Michael Sieg, Erik Schilling, Marco Binder, Thomas Wilhelm Vahlenkamp, Claudia Claus

**Affiliations:** ^1^ Institute of Virology, Faculty of Veterinary Medicine, Leipzig University, Leipzig, Germany; ^2^ Institute of Medical Microbiology and Virology, Medical Faculty, Leipzig University, Leipzig, Germany; ^3^ Institute of Clinical Immunology, Medical Faculty, Leipzig University, Leipzig, Germany; ^4^ Research Group “Dynamics of early viral infection and the innate antiviral response”, Division “Virus-Associated Carcinogenesis”, German Cancer Research Center (DKFZ), Heidelberg, Germany

**Keywords:** 2-deoxy-D-glucose, extracellular acidification rate, extracellular flux analysis, IFNAR, metabolism, oxygen consumption rate, Usutu virus

## Abstract

The mosquito-borne Usutu virus (USUV) is a zoonotic flavivirus and an emerging pathogen. So far therapeutical options or vaccines are not available in human and veterinary medicine. The bioenergetic profile based on extracellular flux analysis revealed an USUV infection-associated significant increase in basal and stressed glycolysis on Vero and with a tendency for basal glycolysis on the avian cell line TME-R derived from Eurasian blackbirds. On both cell lines this was accompanied by a significant drop in the metabolic potential of glycolysis. Moreover, glycolysis contributed to production of virus progeny, as inhibition of glycolysis with 2-deoxy-D-glucose reduced virus yield on Vero by one log_10_ step. Additionally, the increase in glycolysis observed on Vero cells after USUV infection was lost after the addition of exogenous type I interferon (IFN) β. To further explore the contribution of the IFN response pathway to the impact of USUV on cellular metabolism, USUV infection was characterized on human A549 respiratory cells with a knockout of the type I IFN receptor, either solely or together with the receptor of type III IFN. Notably, only the double knockout of types I and III IFN receptor increased permissiveness to USUV and supported viral replication together with an alteration of the glycolytic activity, namely an increase in basal glycolysis to an extent that a further increase after injection of metabolic stressors during extracellular flux analysis was not noted. This study provides evidence for glycolysis as a possible target for therapeutic intervention of USUV replication. Moreover, presented data highlight type I and type III IFN system as a determinant for human host cell permissiveness and for the infection-associated impact on glycolysis.

## Introduction

The mosquito-borne Usutu virus (USUV) is an emerging zoonotic flavivirus (Simmonds et al., 2017) and within the family *Flaviviridae* it belongs to the Japanese encephalitis virus serogroup together with West Nile virus (WNV). USUV is currently circulating in Africa, the Middle East, and Europe (Gill et al., 2020). In multiple European countries its presence was already documented by various methods of detection of viral RNA and of anti-USUV antibodies (Vilibic-Cavlek et al., 2020). Based on phylogenetic analysis this enveloped virus with a single-stranded RNA genome in positive polarity (Simmonds et al., 2017) is arranged in eight lineages, three African (Africa 1 to 3) and five European (Europe 1 to 5) (Cadar et al., 2017; Vilibic-Cavlek et al., 2020). The bird–mosquito–bird transmission cycle comprises a variety of avian species as amplifying hosts and several mosquitoes as vectors, while mammals and humans are considered as dead-end hosts. Despite its high mortality rate in some avian species, in most cases USUV infection in humans is asymptomatic (Benzarti and Garigliany, 2020). However, comparable to encephalitis cases noted for other flaviviruses, neuroinvasion together with the occurrence of neurological symptoms are reported after USUV infection in immunocompromised and even in immunocompetent patients (Pecorari et al., 2009; Grottola et al., 2017; Simonin et al., 2018; Nagy et al., 2019; Zelena et al., 2021).

The threat of evolutionary adaptation together with the potential of alterations of its pathogenicity in humans and/or in its transmission cycle, emphasizes the continuous relevance of the emergence of USUV (Aberle et al., 2018; Roesch et al., 2019). Moreover, WNV and USUV co-circulate and can even simultaneously be present as a cause of neurological symptoms in humans (Vilibic-Cavlek et al., 2014; Nikolay, 2015; Cabanova et al., 2019; Zelena et al., 2021). Thus, the current circulation of USUV in a specific geographic area could serve as an indicator for WNV (Nikolay, 2015). Additionally, some of the virological features and clinical characteristics are similar between USUV and WNV suggesting the study of USUV as a reference for WNV (Nikolay, 2015; Gill et al., 2020). No vaccines or anti-viral treatment options are available so far for USUV disease (Gill et al., 2020). In this regard the viral impact on cellular metabolism could be a promising target with growing importance for the study of new therapeutic intervention options against virus infections (Sumbria et al., 2021). A number of viruses, namely, members of the family *Flaviviridae* cause metabolic alterations to support their productive infection cycle (Sanchez-Garcia et al., 2021; Sumbria et al., 2021). Thus, we explored the USUV infection-associated alterations in the bioenergetic profile in various cell lines. Vero cells are highly susceptible to USUV, which are therefore used for its isolation, cultivation, and titration (Benzarti and Garigliany, 2020). In reflection of its vector-bird transmission cycle and a possible transmission along the respiratory route, we also addressed USUV infection in an avian cell line derived from European blackbirds and in human lung adenocarcinoma A549 cells. We found that USUV infection in Vero was associated with a significant increase in glycolysis. This glycolytic increase supported virus yield and was reduced after application of exogenous interferon (IFN) β to USUV-infected Vero cells. In line with this, the infection on A549 cells was supported in association with an increase in glycolysis after a knockout (KO) of both, type I and III IFN receptors. The data presented highlight glycolysis as a possible target for therapeutic intervention of USUV infection and the types I and III IFN system as a relevant restriction factor for human cell permissiveness to USUV. Thus, surveillance of the development of viral antagonists of the IFN pathway is highly anticipated.

## Material & Methods

### Reagents

The glucose analogue 2-deoxy-D-glucose (2-DG) was purchased from Santa Cruz Biotechnology (Dallas, Texas, USA), IFN β from PeproTech GmbH (Hamburg, Germany) and IFN λ1 from Bio-Techne Corporation (Minneapolis, Minnesota, USA).

### Cultivation of Permanent and Finite Cell Lines

The African green monkey kidney cell line Vero (ATCC CCL-81) and the adenocarcinomic alveolar basal cell line A549 (ATCC CCL-185) were cultivated in high-glucose Dulbecco’s modified Eagle’s medium (DMEM) with 2 mM glutamine, 1% non-essential amino acids (NEAA) and 1% sodium pyruvate. The avian cell line TME-R (FLI CCL V-RIE 1164) was generated from five-day-old whole embryos of the Eurasian blackbird and maintained in equal volumes of Ham’s 12 and Iscove’s modified Dulbecco’s medium. For all cell lines maintenance medium was supplemented with 10% fetal calf serum (FCS) and 100 U/ml penicillin and 100 µg/ml streptomycin and cultivated at 37°C in a humidified atmosphere. All reagents were purchased from Thermo Fisher Scientific, Schwerte, Germany.

A549 KO cell lines were generated by CRISPR/Cas9 technology using lentiviral transduction (lentiCRISPR v2 system, kindly provided by Feng Zhang). Guide RNAs were ordered as dsDNA oligos and cloned into the BsmBI site of the lentiCRISPR v2 plasmid (#52961, Addgene), and lentiviral particles were generated as described before (Wüst et al., 2021). Single cell clones were generated using limiting dilution.

### USUV Strains

USUV strains Africa 3 (KY294723.1) and Europe 3 (KY199558.1) were isolated from avian organ samples kindly provided by the Saxon State Laboratory of Health and Veterinary Affairs (Leipzig, Germany). Briefly, small amounts of selected tissues (spleen, liver, kidney, and brain) were dissolved in DMEM, homogenized, centrifuged and incubated on Vero cells for virus isolation. Sequencing of a gene segment encoding USUV envelope protein and phylogenetic analysis by NCBI’s Basic Local Alignment Search Tool (BLAST) were used for phylogenetic characterization of USUV isolates.

### USUV Infection

USUV strains were used at passage 2 after initial isolation. For infection an multiplicity of infection (MOI) of 0.1 and an MOI of 1 were employed. After incubation for 2 h and twice PBS washing, medium change to maintenance medium containing 2% FCS and 100 U/ml penicillin and 100 µg/ml streptomycin was carried out.

### USUV Titer Determination and Immunofluorescence Analysis

Titer determination was performed on Vero cells overlaid with 1% carboxymethylcellulose followed by incubation for 3 days. After fixation with 2% (v/v) formaldehyde solution at room temperature for 30 min immunostaining was accomplished using flavivirus-specific monoclonal primary antibody (mouse anti-pan-flavivirus 3571 antibody, Santa Cruz Biotechnology, Dallas, Texas, USA) diluted 1:800 in permeabilization/wash solution containing 0.1% (w/v) BSA and 0.1% (w/v) saponine in PBS for incubation at 4°C overnight. Secondary antibody Alexa fluor 546 goat anti-mouse (Thermo Fisher Scientific, Schwerte, Germany) was diluted 1:1,000 in permeabilization/wash solution with the nuclear dye 4′,6-diamidino-2-phenylindole (DAPI, Carl Roth, Karlsruhe, Germany) at 100 µg/ml followed by an incubation for 1 h at 37°C. Immunofluorescence analysis was carried out accordingly. As a means of quantification, four images of random microscopic fields were subjected to counting of USUV antigen-positive cells to determine % of infected cells. For titer determination, focus-forming units (FFU) were determined by counting of foci by fluorescence microscopy.

### Metabolic Flux Measurement, Analysis of the Metabolic Phenotype and the Metabolic Potential

Real-time measurement of metabolic activity was performed by the XFp extracellular flux analyzer (Agilent Seahorse Technologies, Santa Clara, CA, USA). The oxygen consumption rate (OCR) is proportional to mitochondrial respiration (oxidative phosphorylation). Extracellular acidification rate (ECAR) refers to changes in the pH and as such is proportional to glycolysis (Ferrick et al., 2008). Cells were plated into XFp miniplates at 1.6 × 10^4^ cells per well and measured by the cell energy phenotype test kit based on co-injection of the ATP synthase inhibitor oligomycin (1 µM) and trifluoromethoxy carbonylcyanide phenylhydrazone (FCCP, 0.8 µM) (Agilent Seahorse Technologies, Santa Clara, CA, USA). FCCP-induced depolarization of the mitochondrial membrane leads to an increase in OCR as a compensation of the loss in the mitochondrial membrane potential. The inhibition of the ATP synthase with oligomycin results in compensation of the loss of energy supply through glycolysis. All metabolic measurements were conducted in duplicates per sample (n = 2, experimental replicates) and repeated in three independent experiments (n = 3, biological replicates). Prior to measurement cultivation medium was changed to XF DMEM (Agilent Seahorse Technologies, Santa Clara, CA, USA) supplemented with 10 mM glucose (Agilent Seahorse Bioscience), 1 mM sodium pyruvate and 2 mM L-glutamine (Thermo Fisher Scientific, Schwerte, Germany). Obtained data was used for calculation of metabolic potential as the capacity of a cell to respond to a rise in the cellular energy demand is based on the increase of stressed (after injection of the inhibitors) OCR and ECAR over basal (baseline) OCR and ECAR, respectively. As an additional means of calculation of metabolic changes under a given condition, the ratio of basal OCR and ECAR (the OCR/ECAR ratio) based on values obtained at measurement point three was calculated. The lower the OCR/ECAR value, the higher is the cellular reliance on glycolysis (Zhang et al., 2012). Normalization was based on protein content, which was determined as optical density (OD) by Bradford assay to calculate OCR and ECAR values as (pmol/min)/OD and (mpH/min)/OD, respectively.

### Cell Viability Assay

Assessment of cytotoxicity was performed in triplicate in 96-well plates using Rotitest vital assay (Carl Roth, Karlsruhe, Germany) according to manufacturer’s instructions. This assay is based on the conversion of WST-8 tetrazolium salt into colorimetric formazan by cellular enzymes. Briefly, 1 to 4 h after application of 1:10 WST-8 substrate per well the OD was measured at 450 nm at ELISA reader. Cell viability was defined as percentage of control.

### Western Blot Analysis

Cell lysis was carried out with RIPA buffer (50 mM Tris, 150 mM NaCl, 1% Nonidet P-40, 0.5% deoxycholate, 0.1% SDS; pH 7.5) supplemented with complete EDTA-free protease inhibitor cocktail (Roche Diagnostics, Mannheim, Germany) and with phosphatase inhibitors (1 mM Na_3_VO_4_ and 50 mM NaF) followed by sonication on ice. Cell lysates (25–30 µg according to protein concentration determination by DC Protein Assay (Bio-Rad Laboratories (Hercules, CA, USA) were boiled in 1× Laemmli sample buffer and run on a 12% SDS-polyacrylamide gel (Protean II, Bio-Rad GmbH) and transferred to polyvinylidene difluoride (PVDF) membranes (Amersham Biosciences, Munich, Germany). PVDF membranes were blocked with 5% milk powder and probed with the following antibodies (Ab): anti-phospho-STAT1 rabbit Ab (Tyr701, D4A7, 1:1,000, Cell Signaling, Danvers, MA, USA), and anti-β-actin mouse Ab (clone AC 74, 1:2,000, Sigma-Aldrich, St. Louis, USA). Primary antibodies were detected with the following POD-conjugated secondary antibodies: goat anti-rabbit IgG Ab (1:20,000, Dianova, Hamburg, Germany) or goat anti-mouse IgG Ab (1:8,000, Sigma-Aldrich, St. Louis, USA). Chemiluminescent detection on PVDF membranes was carried out with ECL-A/ECL-B substrate (Sigma-Aldrich, St. Louis, USA) on a Luminescent Image Analyzer (LAS 1000, Fujifilm, Tokyo, Japan).

### Statistical Analysis

Data was analyzed by paired t-test and one-way analysis of variance (ANOVA) and Dunnett’s *post-hoc* multiple comparisons with GraphPad Prism software 9 (San Diego, USA). Data are shown as the mean ± standard deviation (SD) except for extracellular flux measurement displaying the mean ± standard error of the mean (SEM). Statistical significance is indicated as *p <0.05, **p <0.01, ***p <0.001, ****p <0.0001.

## Results

### A High Infection Rate of USUV on Vero Cells Is Associated With an Increase in Glycolysis

**Figure 1:**
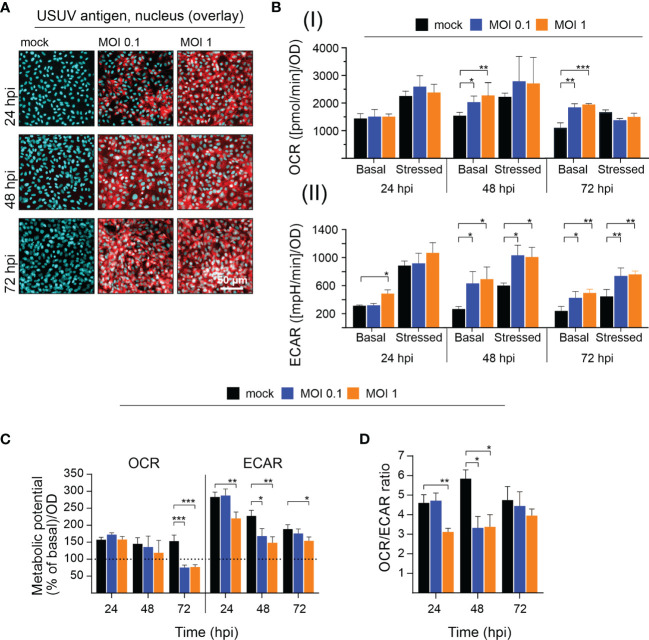
Extracellular flux measurement indicates an increase in glycolytic activity in Vero cells after infection with USUV. A low (of 0.1) and a high (of 1) MOI were applied to address the influence of USUV load on **(A)** infectivity rate and **(B–D)** metabolic activity in Vero cells. **(A)** For indicated time points the number of infected Vero cells was qualitatively assessed by fluorescence microscopy after immunofluorescence analysis with pan-flavivirus antibody (shown in red as a representative for n = 3 together with nuclear counterstain DAPI in blue). **(B)** Quantification (mean ± SD, n = 3) of **(I)** OCR and **(II)** ECAR under basal and stressed (after co-injection of inhibitors and thus under induced energy demand) conditions. **(C)** The metabolic potential was determined through the percent increase in stressed OCR and ECAR over basal OCR and ECAR. **(B, C)** Calculations were performed by the energy phenotype test report generator software. **(D)** OCR/ECAR ratio was calculated based on basal OCR and ECAR as determined at measurement point 3 of extracellular flux measurements. **(B–D)** Data are shown as mean ± SD (n = 3). Statistical significance is indicated as *p < 0.05, **p < 0.01, and ***p < 0.001, performed by ANOVA and Dunnett’s *post-hoc* multiple comparisons test.

Due to their high permissiveness for USUV (Bakonyi et al., 2005; Kuchinsky et al., 2020) Vero cells were selected in initial experiments for the analysis of the impact of USUV on cellular metabolism. A low (of 0.1) and high (of 1) MOI were employed to account for the influence of the initial infection rate. At 96 hpi a cytopathic effect (CPE) was induced by both MOIs, which results in alterations of the Vero cell monolayer (**Supplement Figure 1A**). Extracellular flux analysis measures metabolic activity in living cells. Thus, at least a 50% rate of infected cells within the cell monolayer was anticipated to assess virus infection-associated metabolic alterations. The number of infected cells was semiquantitatively determined by immunofluorescence assay. The immunofluorescence analysis shown in **Figure 1A** reflects the anticipated high level of susceptibility of Vero cells to USUV. Already at 24 h post-infection (hpi) almost all cells were infected at MOI 1 and at 48 hpi no difference in the rate of USUV-positive cells was notable between MOI 0.1 and 1. Next, these infection kinetics were analyzed by extracellular flux analysis with the cell energy phenotype test kit. The rate of mitochondrial respiration (oxidative phosphorylation) was measured through OCR and the rate of glycolysis through ECAR. This allowed for characterization of the metabolic phenotype and of the capacity of USUV-infected cells in comparison to the mock infection responding to a higher energy demand after co-injection of two metabolic stressors, FCCP, and oligomycin. **Supplement Figure 1B** displays the graphical reflection of OCR and ECAR over a total time period of 50 min. Three measurement points of basal metabolic activity were followed by co-injection of the inhibitors and measurement of the stressed phenotype. **Supplement Figure 1B** indicates a shift in basal and stressed metabolic activity in Vero cells after USUV infection starting at 24 hpi for MOI 1 with a peak at 48 and 72 hpi for both MOIs. The calculation of basal and stressed OCR highlights a significant alteration in OCR and as such in mitochondrial respiration after USUV infection only at basal level at 48 and 72 hpi (**Figure 1B**). In contrast to OCR, significant alterations in ECAR were detected at 48 and 72 hpi for both MOIs and for both conditions, basal and stressed ECAR. A significant increase in ECAR was already indicated at 24 hpi, but only for the higher MOI 1 and only for basal ECAR (**Figure 1B**). Based on these observations, we determined the metabolic potential as the capacity of a cell to meet the increase in energy demand after application of the metabolic stressors through an increase in mitochondrial respiration and glycolysis. A significant drop in the capacity of Vero to meet the increased energy demand of the stressed phenotype through glycolysis was detected for MOI 1 at 24 hpi and for both MOIs at 48 hpi after USUV infection (**Figure 1C**). This indicates that the detected increase (**Figure 1B**) in basal glycolysis in USUV-infected Vero cells is close to the maximal glycolytic rate. Uninfected Vero cells possess a higher metabolic potential than USUV-infected Vero, which suggests that during USUV infection Vero cells undergo metabolic exhaustion. Furthermore, the shift to a higher glycolytic activity at basal level was also reflected by a significant decrease in OCR/ECAR ratio in Vero at 24 hpi for MOI 1 and at 48 hpi for both MOIs (**Figure 1D**). In summary, extracellular flux analysis of USUV-infected Vero cells under basal and stressed conditions revealed a significant increase in ECAR indicative for an infection-associated induction of glycolytic activity.

### Glycolysis Supports Virus Yield During USUV Infection of Vero Cells and is Influenced by Exogenous Interferon Beta

**Figure 2:**
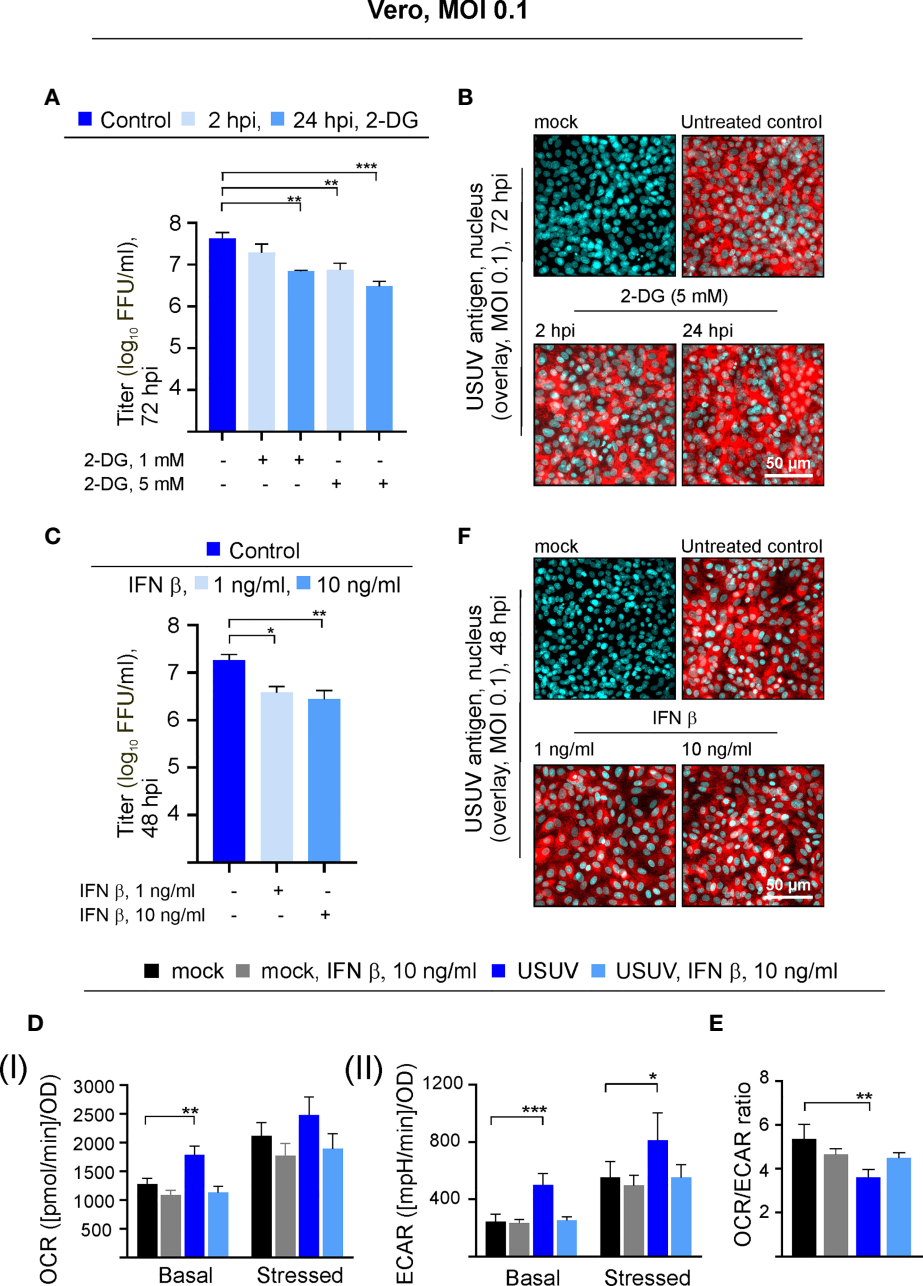
The USUV-associated increase in glycolysis supports USUV yield and is lost in the presence of IFN β. Experiments on Vero cells were conducted at an MOI of 0.1. The dose- and application time point-dependent influence of the glycolysis inhibitor 2-DG on USUV yield was determined by **(A)** focus-forming assay and **(B)** immunofluorescence analysis of USUV-infected Vero cells with pan-flavivirus antibodies (shown in red) and nuclear counterstain (shown in blue) at 72 hpi after application of 5 mM 2-DG at 2 and 24 hpi. The impact of 1 and 10 ng/ml IFN β applied at 24 hpi to USUV-infected Vero cells was analyzed at 48 hpi by focus-forming assay **(C)** and immunofluorescence analysis **(F)**. **(D)** At 48 hpi basal and stressed **(I)** OCR and **(II)** ECAR values were determined through extracellular flux measurements with the cell phenotype test kit under basal and stressed conditions (co-application of 0.8 µM FCCP and 1 µM oligomycin) in the presence of 10 ng/ml IFN β applied at 24 hpi. **(E)** OCR/ECAR ratio was calculated based on basal OCR and ECAR as determined at measurement point 3 of extracellular flux measurements. **(A, C–E)** Data (n = 3) are shown as mean ± SD involving statistical analysis for **(A, C)** in comparison to solvent control-treated samples and **(D, E)** in comparison to the mock-infected control performed by ANOVA and Dunnett’s *post-hoc* multiple comparisons test. Statistical significance is calculated as *p < 0.05), **p < 0.01, and ***p < 0.001.

USUV infection appears to induce a shift of cellular metabolism to a higher glycolytic activity in Vero cells (**Figure 1B**). Based on the hypothesis that glycolysis could support USUV replication cycle, the glucose analogue 2-DG as an inhibitor of glycolysis was applied to USUV-infected Vero cells. An MOI of 0.1 was employed as a lower initial infection rate was anticipated to display a higher sensitivity to the application of inhibitors. The addition of 2-DG at two different concentrations (1 and 5 mM) and two different time points (2 and 24 hpi) reduced extracellular USUV particles significantly in comparison to the untreated control at 72 hpi (**Figure 2A**). However, viral titers in supernatants of 2-DG-treated cells collected at 24 and 48 hpi were comparable to the untreated control (data not shown). With one log_10_ reduction in virus titer the highest effect was detected after application of 5 mM 2-DG at 24 hpi. Viability assessment of 2-DG treatment of Vero cells revealed no cytotoxic effects by this compound, which could otherwise affect virus replication and thus virus particle production (**Supplement Figure 2A**). Immunofluorescence staining at 72 hpi was similar between control- and 2-DG (5 mM at 2 hpi and 24 hpi)-treated USUV-infected cultures (**Figure 2B**), which indicates that 2-DG treatment did not alter the number of USUV-positive cells.

Vero cells contain a homozygous deletion on chromosome 12, which resulted in the loss of the type I IFN genes α and β as shown by DNA hybridization techniques (Diaz et al., 1988) and whole-genome sequencing (Osada et al., 2014). They are able to respond to exogenous type I IFN, but they are not able to produce type I IFNs during a virus infection. This led us to the investigation of the metabolic activity in USUV-infected Vero cells in the presence of exogenous type I IFN β. Exogenous IFN β (1 and 10 ng/ml) was added at 24 hpi, as metabolic alterations in association with USUV infection started at 24 hpi and were highly pronounced at 48 hpi. Thus, antiviral and antiproliferative effects by IFN β should be detectable at 48 hpi after IFN β application at 24 hpi. As an antiviral effect on USUV infection, application of IFN β reduced extracellular virus titer at 48 hpi significantly, which was comparable between the applied IFN β concentrations (**Figure 2C**). Hereafter, the cell energy phenotype test was performed assessing the impact of exogenous IFN β (added at 24 hpi at a concentration of 10 ng/ml) on metabolic activity of USUV-infected Vero cells (**Figure 2D**). The graphical representation of the extracellular flux measurements at 48 hpi is provided in **Supplement Figure 2B**. As noted before (**Figure 1**), in the presence of USUV basal OCR and basal and stressed ECAR were significantly increased in comparison to the mock control. A notable and significant reduction in basal and stressed metabolic activity occurred after the addition of IFN β to USUV-infected cells (**Figure 2D**). Here, metabolic activity was highly similar to that of the mock-infected control. Regarding mock infection only a slight influence on metabolic activity was detected after the addition of exogenous IFN β. The calculation of OCR/ECAR ratio revealed two aspects. First, in agreement with the observed reduction in basal OCR and the unaffected basal ECAR (**Figure 2D** and **Supplement Figure 2B**) a slight reduction in this ratio occurred after the addition of IFN β to mock-infected cells (**Figure 2E**). Second, the significant reduction in the OCR/ECAR ratio of USUV-infected Vero cells as indicative for an increased reliance on glycolysis was lost in the presence of exogenous IFN β. Hereafter immunofluorescence staining as indicative for the number of USUV-infected cells was performed at 48 hpi. No differences between control and IFN β-treated cells were detected (**Figure 2F**). Thus, the observed metabolic alterations after IFN β treatment (**Figure 2D**) were not associated with differences in the number of infected cells within the cell monolayer. Together our data point to a supportive role of glycolysis during USUV infection in Vero cells. Additionally, the infection-associated increase in glycolysis on Vero was reduced after application of exogenous IFN β.

### The Avian Cell Line TME-R Supports High-Level Infection of USUV

**Figure 3:**
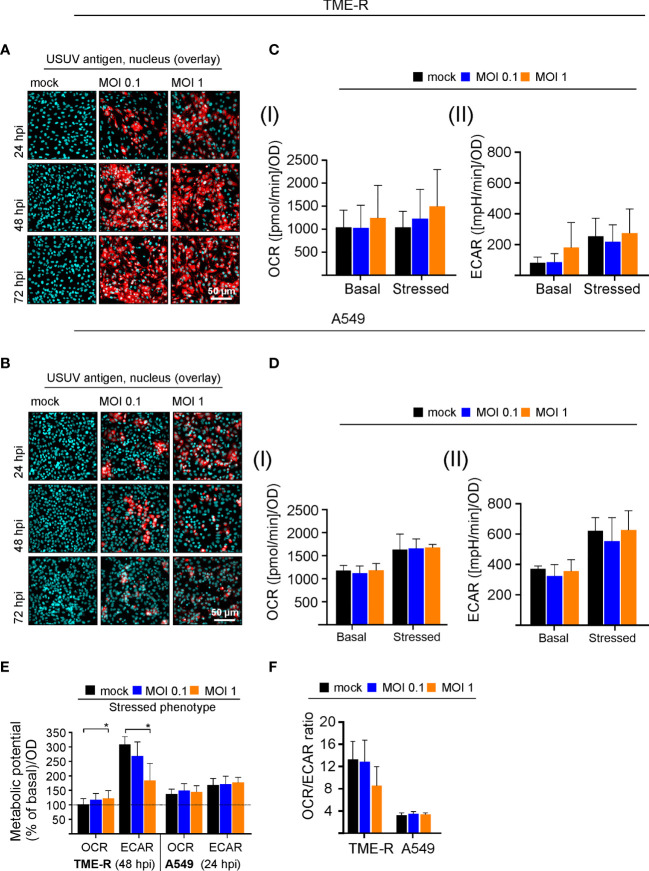
Analysis of the cell-type specificity of USUV infection rate and the associated metabolic impact. **(A, B)** The onset of maximum rate of infected cells was determined by immunofluorescence analysis for **(A)** TME-R and **(B)** A549 cells with pan-flavivirus antibodies for low (MOI 0.1) and high (MOI 1) infectivity rate (representatively shown in red for n = 3 with nuclear counterstain in blue). **(C)** At 48 hpi for TME-R and **(D)** at 24 hpi for A549 cells mitochondrial respiration based on **(I)** OCR values and glycolysis based on **(II)** ECAR values were determined through extracellular flux measurements under basal (without treatment) and stressed conditions (co-application of 0.8 µM FCCP and 1 µM oligomycin). **(E)** The metabolic potential was determined through the percent increase in stressed OCR and ECAR over basal OCR and ECAR. **(F)** OCR/ECAR ratio was calculated based on basal OCR and ECAR as determined at measurement point 3 of extracellular flux measurements. Data are shown as mean ± SD (n = 3). Statistical significance is indicated as *p < 0.05, performed by ANOVA and Dunnett’s *post-hoc* multiple comparisons test.

The initial observations on USUV infection-associated alterations in metabolic activity in Vero cells were extended to two additional cell lines to address cell type-specific aspects and its multi-host replication cycle. As a representative of its avian host the TME-R cell line derived from embryos of Eurasian blackbirds was used. In reflection of the susceptibility of the epithelium of the upper human respiratory tract to flaviviruses (Vielle et al., 2019) and its relevance for the transmission cycle to humans, we have included A549 cells as type II pulmonary epithelial cell culture model. Immunofluorescence analysis revealed differences in the course of infection between TME-R and A549 cells and between the applied MOIs on A549 cells (**Figures 3A, B**). On TME-R an infectivity rate of 77% ± 1.5% (MOI 1) and 65.1% ± 18.6% (MOI 0.1) was reached at 24 hpi and at 48 hpi an infectivity rate exceeding 90% was detected for both MOIs. Thus, for TME-R 48 hpi as the earliest time point of the onset of a high infection rate on TME-R was chosen for extracellular flux measurement of USUV- and mock-infected TME-R. On A549 cells immunofluorescence analysis revealed no further increase in USUV antigen-positive cells between 48 and 72 hpi in comparison to 24 hpi. Quantification of the number of USUV-antigen positive A549 cells as indicated by immunofluorescence analysis revealed an infectivity rate of 55.4% ± 2.8%, 24.7% ± 6.8%, 21.2% ± 8.1% (MOI 1) and 25.4% ± 10.9%, 23.9% ± 8.3%; 21.9% ± 3.3% (MOI 0.1) at 24, 48, and 72 hpi, respectively. The 24 hpi time point was subjected to metabolic assessment, as this time point had an infectivity rate of at least 50%. The graphical representation of the extracellular flux measurements is shown in **Supplement Figure 3**. **Figure 3C** highlights that TME-R cells have a tendency to increase in glycolysis as revealed by the increase in basal ECAR values after infection with USUV at an MOI of 1. However, this slight increase was associated with a distinctive standard deviation. In comparison to the mock control, ECAR values for basal glycolytic activity after USUV infection at an MOI of 1 were increased by 94.6% ± 73.8% in comparison to the mock control. Under all addressed conditions USUV-infected A549 cells were comparable to the uninfected control (**Figure 3D**).

Visualizing the impact of USUV infection on cellular metabolism and to compare its influence between the addressed cell lines, the metabolic potential (**Figure 3E**) and the ratio of basal OCR and basal ECAR (**Figure 3F**) were analyzed as it was shown for Vero cells in **Figures 1C, D**. A significant drop in the capacity of TME-R to meet the increased energy demand of the stressed phenotype through glycolysis was detected after USUV infection (**Figure 3E**). This tendency of TME-R to a higher basal glycolytic activity (**Figure 3C**) was also reflected by a drop in OCR/ECAR ratio in USUV-infected TME-R (**Figure 3F**). This decrease in OCR/ECAR ratio was comparable to USUV-infected Vero cells (**Figure 1D**). Both parameters, the cell energy phenotype and the OCR/ECAR ratio point to a higher glycolytic activity after infection of TME-R with USUV at an MOI of 1 and highlight a significant shift of cellular metabolism to glycolysis in Vero. In summary, TME-R support high-level infection of USUV, whereas A549 are indicative for an impaired infection kinetics. TME-R demonstrate the tendency of a glycolytic impact of USUV infection.

### Interferon Signaling Influences Permissiveness and Metabolic Response of A549 Cells to USUV Infection

**Figure 4:**
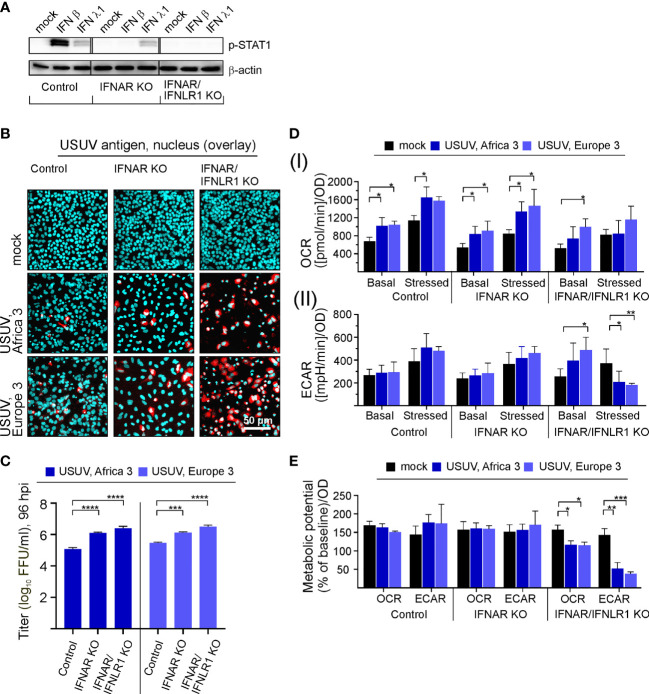
Type I and III IFN signaling influences permissiveness of A549 cells to USUV infection-associated metabolic alterations. **(A)** Western blot analysis of A549 cells with KO of the type I IFN receptor IFNAR either solely or together with the type III IFN receptor (IFNLR1) and A549 control cells. Antibodies against phosphorylated STAT1 were used for analysis of samples with exogenous IFN β (10 ng/ml) or IFN λ1 (100 ng/ml) after an incubation for 24 h. **(B–E)** Infection with USUV strain Africa 3 and Europe 3 at MOI 0.1 was analyzed at 96 hpi. **(B)** Immunofluorescence analysis with pan-flavivirus antibodies (representatively shown in red for n = 3 with nuclear counterstain in blue). **(C)** Virus yield was determined by focus-forming assay. **(D)** Extracellular flux measurement with the cell energy phenotype test kit to determine **(I)** OCR and **(II)** ECAR values at basal and stressed (after co-application of 0.8 µM FCCP and 1 µM oligomycin) conditions. **(E)** The metabolic potential was determined through the percent increase in stressed OCR and ECAR over basal OCR and ECAR. **(C–E)** Data (n = 3) are shown as mean ± SD involving statistical analysis in comparison to the control performed by ANOVA and Dunnett’s *post-hoc* multiple comparisons test. Statistical significance is shown as *p < 0.05, **p < 0.01, ***p < 0.001) and ****p < 0.001).

Based on the effect of exogenous IFN β on USUV infection in Vero cells, we hypothesized the IFN pathway may contribute to the infection in A549 cells. Investigating the IFN-metabolism interaction in more detail, we subjected A549 cells with a CRISPR-Cas-mediated KO of the type I IFN receptor IFNAR either solely (IFNAR KO) or in conjunction with type III IFN receptor IFNLR1 (IFNAR/IFNLR1 KO) to USUV infection. This set-up analyzed the aspect of USUV strain specificity through the inclusion of USUV strain Europe 3 in addition to Africa 3 strain. As representatively shown for application of exogenous type I IFN β (10 ng/ml) and type III IFN λ1 (100 ng/ml), phosphorylation and thus activation of signal transducer and activator of transcription 1 (STAT1) as an essential transcription factor within the IFN signaling pathway was absent in the IFNAR KO and the IFNAR/IFNLR1 KO cells, respectively (**Figure 4A**). The application of IFN β confirmed the absence of p-STAT1 on IFNAR KO A549, whereas signaling through IFNLR1 after IFN λ1 application was detectable as indicated by p-STAT1. On IFNAR/IFNLR1 KO no p-STAT1 signal was detectable after IFN β and IFN λ1 application. This shows that p-STAT1 is induced by IFN λ and as reviewed by Stanifer and colleagues IFN β and IFN λ1 can induce a similar pattern of ISGs, but this induction can occur at a different time scale (Stanifer et al., 2020). The IFNAR/IFNLR1 KO A549 cells enable the analysis of both IFN types on cellular metabolism during USUV infection. Monitoring the effect of the IFN receptor KO on USUV, an MOI of 0.1 with an extended incubation time frame to 96 h was selected. This time frame was sufficient for the development of a CPE (**Supplement Figure 4A**). Moreover, a high proportion of positive signals in the immunofluorescence analysis of A549 IFNAR/IFNLR1 double KO cells (**Figure 4B**) was detected in addition to a significant increase in virus titer in IFNAR KO and IFNAR/IFNLR1 double KO cells compared to control (**Figure 4C**). As a next step, extracellular flux measurement was performed (**Supplement Figure 4B**). Regarding OCR significant increases were noted after USUV infection in A549 control, IFNAR KO and partly in IFNAR/IFNLR1 double KO cells in comparison to the mock-infected controls (**Figure 4D**). In contrast only in IFNAR/IFNLR1 double KO cells a significant increase in basal glycolysis of USUV strain Europe 3 and a strain-independent significant reduction in stressed ECAR occurred after USUV infection (**Figure 4D**). Vero cells are not able to generate type I IFNs and as such lack IFN signaling. For comparison of the metabolic impact of USUV on Vero cells with A549 control and receptor KO cells, percent metabolic activity after USUV infection was determined in reference to the mock control (set to 100%) (**Supplement Figure 5**). Similar to Vero cells, A549 cells with IFNAR/IFNLR1 KO were increased in basal glycolysis after USUV infection. However, in contrast to Vero cells this was accompanied by a decrease in stressed glycolysis after injection of the metabolic stressors oligomycin and FCCP. This could be associated with their tumorigenic origin and their reliance on glycolysis. This somehow exhaustive effect of USUV on glycolysis of A549 cells with IFNAR/IFNLR1 KO was also present through a significant drop in the ECAR metabolic potential (**Figure 4E**), which was also noted after infection of Vero cells (**Figure 1C**). In conclusion, in the absence of both, type I and III IFN signaling, USUV infection of A549 as alveolar epithelial cells was associated with an alteration in glycolysis.

## Discussion

USUV is an emerging pathogen with a continuous spread across Europe. The similarity of its transmission and genetic characteristics to WNV highlights not only the possibility of USUV to further evolve as a more severe human pathogen. Moreover, USUV as a biosafety 2 pathogen could serve as a role model for the biosafety 3 WNV. With our study we add three important and novel aspects to USUV pathogenicity. First, the USUV infection-associated cell-specific increase in glycolytic activity supports USUV infection. Second, type I and III IFNs influence the productive infection of USUV in human cells. Third, the USUV infection-dependent increase in glycolysis is counteracted by the IFN response.

The high USUV-permissiveness of the TME-R cell line is in agreement with the *in vivo* susceptibility of Eurasian blackbirds (Giglia et al., 2021). Additionally, we have also addressed the potential adaptation of USUV to lung carcinoma A549 cells as a commonly used type II pulmonary epithelial cell model. The occupationally acquired WNV infection among turkey breeder farm workers suggests the potential of a flavivirus transmission cycle *via* aerosols (Centers for Disease and Prevention, 2003). As indicated by our results on A549 cells after KO of type I and III IFN receptors, the infection of respiratory cell types can be highly productive in the absence of IFN signaling. Further adaptation of USUV could lead to refinement of viral antagonists of the human IFN response. In line with our notification on a limited susceptibility of A549 cells to USUV, USUV had a lower replication and production rate of extracellular virus particles on primary human nasal epithelial cells (NECs) as a representative cell model for the human upper respiratory tract epithelium than WNV (Vielle et al., 2019). The time course of the IFN response on A549 cells could account for the drop in the number of infected cells after 24 hpi, as it was shown for human HEp-2 cells that the type I and III IFNs were only present in the supernatant at 24 hpi (Scagnolari et al., 2013).

The general relevance of the antiviral countermeasures posed by the IFN system against flaviviruses is reflected by their multiple exploited mechanisms to counteract IFN signaling as outlined in a recent review (Miorin et al., 2017). These mechanisms include antagonism of RIG-I like receptors (RLR) and also of components downstream of pattern-recognition receptors (PRRs). For example, the NS5 protein of WNV binds to cellular prolidase as a regulatory component of IFNAR expression, which leads to downregulation of IFNAR expression and as such to impairment of ISG expression (Lubick et al., 2015). Additionally, WNV strategies to avoid IFN signaling include blocking JAK1 and TYK2 activation and as such interference with downstream STAT1 phosphorylation and nuclear translocation (Guo et al., 2005; Liu et al., 2005). Similar to our observations for impaired infection of USUV on A549 cells, virus titer of a NS2A/A30P (alanine 30-to-proline) mutant WNV dropped over time of cultivation together with the absence of a CPE otherwise noted for wild type (WT) WNV (Liu et al., 2006). This A30P mutation in the viral NS2A abolished viral interference with IFN β transcription. The study by Liu et al. does also provide another set of data in support of the hypothesis of the relevance of both, type I and III IFNs during USUV infection. The infection of AG129 mice with a KO of the IFN α/β/γ receptors with the NS2A/A30P mutant WNV was originally anticipated to be comparable to WT virus. However, NS2A/A30P mutant WNV was still attenuated with a significant difference in viremia to WT WNV, suggesting that the attenuation of this mutant strain was only partially attributable to IFN α/β response. Our data on A549 IFNAR/IFNLR KO suggest that one of the additional factors could be type III IFN signaling. Furthermore, mouse models highlight the restrictive nature of type I and III IFN signaling on USUV: while adult WT mice are not susceptible with only a very limited development of pathogenesis (Blazquez et al., 2015), the KO of the type I IFN receptor (*ifnar1^−/−^*) was sufficient to confer a high level of susceptibility and mortality (Martin-Acebes et al., 2016). With our study we highlight avian TME-R together with human A549 cells with IFN receptor KO as a suitable cell culture model for USUV pathogenesis in complementation of *ifnar1^−/−^* mice (Kuchinsky et al., 2020). The use of human IFN receptor KO cells could contribute to the identification of human IFN-induced genes (ISGs) with antiviral activity against USUV.

The analysis of cellular metabolism during virus infections could reveal cell specific factors that are relevant for the interpretation of the course of infection on this cell line. Over time of USUV infection on Vero cells the increase in OCR and ECAR over the mock control slows down. This could contribute to the drop in the growth kinetics reported by Kuchinsky and others for MOI 0.1 on Vero at 96 hpi (Kuchinsky et al., 2020). Additionally, the reported peak at 48 hpi of about 8 log_10_ plaque forming units per ml coincides with the peak of metabolic alterations on Vero as shown by our study. Moreover, the onset of a cytopathic effect (**Supplement Figure 1A**) was associated with a significant drop in the metabolic potential facing artificial energy demand by OCR at 72 hpi assuming the initialization of metabolic exhaustive events under USUV infection. A549 cells with the KO of type I and III IFNs appear to be comparable to Vero cells in the course of USUV infection, which in addition to the lack of generation of type I IFNs were suggested to have a limited susceptibility to exogenous type III IFNs (Scagnolari et al., 2013). The analysis of Africa 3 and Europe 3 as two of the currently in Europe circulating USUV strains (Michel et al., 2019) on A549 cells with the IFNAR/IFNLR1 KO revealed similar metabolic effects. Thus, the infection-associated metabolic alterations, namely the observed increase in glycolysis could be conserved among USUV strains. An impact on cellular metabolism is for example described for the related Dengue virus: the infection of human foreskin fibroblasts results in an increase in glucose transporter protein levels and in hexokinase 2 mRNA and protein levels (Fontaine et al., 2015). With our study we add an increase in glycolysis as a new aspect to the association of the USUV and possibly flavivirus replication cycle with host cell metabolism. So far the association with lipid metabolism was described for several flaviviruses including WNV (Mackenzie et al., 2007). This includes the identification of acetyl-CoA carboxylase as a potential target for pharmaceutical intervention with USUV and WNV infection (Merino-Ramos et al., 2016).

The IFN–glycolysis axis as identified by our study is also relevant in the antiproliferative impact of IFN as recently highlighted by the reduction of the metabolic glycolytic response of murine bone marrow-derived macrophages to infection with *Mycobacterium tuberculosis* after application of IFN β (Olson et al., 2021). Further studies are required to elucidate the co-operative antiviral activity of type I and III IFNs against USUV and the relevance of type I and III IFNs for the anti-proliferative action of the IFN system during virus infections. Type III IFNs are especially present at respiratory mucosal surfaces (Stanifer et al., 2020) for which A549 as addressed in our study are a representative cell type.

Our study suggests the continuous surveillance of the presence of viral IFN antagonists in USUV strains as type I and III IFNs are important human restriction factors against USUV. So far mostly asymptomatic or mild cases are noted for human infection, but encephalitis and meningoencephalitis can occur as severe complications not only in immunocompromised patients (Pecorari et al., 2009; Cavrini et al., 2011; Pacenti et al., 2019). The *in vivo* clinical data on neuropathogenicity is supported by its replication in iPSCs-derived human neural stem cells (Riccetti et al., 2020). Here we highlight the relevance of metabolic alterations and as such glycolytic activity during USUV infection and their implication in antiviral countermeasures of the IFN system. In the context of the identification of antiviral therapeutical options, the impairment of viral replication in a rhinovirus-infected murine model (Gualdoni et al., 2018) and the curation of human genital herpes infection with 2-DG (Blough and Giuntoli, 1979) highlight the efficacy of 2-DG against virus infections. Clinically approved glycolysis inhibitors might be a supportive antiviral treatment option for USUV infection and potentially for flavivirus infections in general.

## Data Availability Statement

The original contributions presented in the study are included in the article/**Supplementary Material**. Further inquiries can be directed to the corresponding author.

## Author Contributions

Conceptualization, MW, MS, CC. Formal analysis, investigation, MW, ES. Methodology, MW, MS, MB, and CC. Supervision, MS and CC. Resources, TV. Writing original draft, MW and CC. All authors listed have made a substantial, direct, and intellectual contribution to the work and approved it for publication.

## Funding

This project was funded by the Dres. Jutta & GeorgBruns-Stiftung during a scholarship for innovative veterinary medicine. We acknowledge support from Leipzig University for Open Access Publishing.

## Conflict of Interest

The authors declare that the research was conducted in the absence of any commercial or financial relationships that could be construed as a potential conflict of interest.

## Publisher’s Note

All claims expressed in this article are solely those of the authors and do not necessarily represent those of their affiliated organizations, or those of the publisher, the editors and the reviewers. Any product that may be evaluated in this article, or claim that may be made by its manufacturer, is not guaranteed or endorsed by the publisher.
