# Supplementary material for: The Interferon Response Dampens the Usutu Virus Infection-Associated Increase in Glycolysis
Source: Front Cell Infect Microbiol. 2022 Feb 4;12:823181. doi: 10.3389/fcimb.2022.823181 (PMC8855070; doi:10.3389/fcimb.2022.823181)
Supplement: Supplementary file 1 [file DataSheet_1.docx]

Supplementary material to

**The interferon response dampens the Usutu virus infection-associated increase in glycolysis**

Maria Elisabeth Wald^1, 2^, Michael Sieg^1^, Erik Schilling^3^, Marco Binder^4^, Thomas Wilhelm Vahlenkamp^1^, Claudia Claus^2*^

^1^ Institute of Virology, Faculty of Veterinary Medicine, Leipzig University, An den Tierkliniken 29, 04103 Leipzig, Germany

^2^ Institute of Medical Microbiology and Virology, Medical Faculty, Leipzig University, Johannisallee 30, 04103 Leipzig, Germany

^3^ Institute of Clinical Immunology, Medical Faculty, Leipzig University, Johannisallee 30, 04103 Leipzig, Germany

^4^ Research Group "Dynamics of early viral infection and the innate antiviral response", Division "Virus-Associated Carcinogenesis", German Cancer Research Center (DKFZ), 69120 Heidelberg, Germany

***** Correspondence: claudia.claus@medizin.uni-leipzig.de; Tel.: +49 341 9714321

**
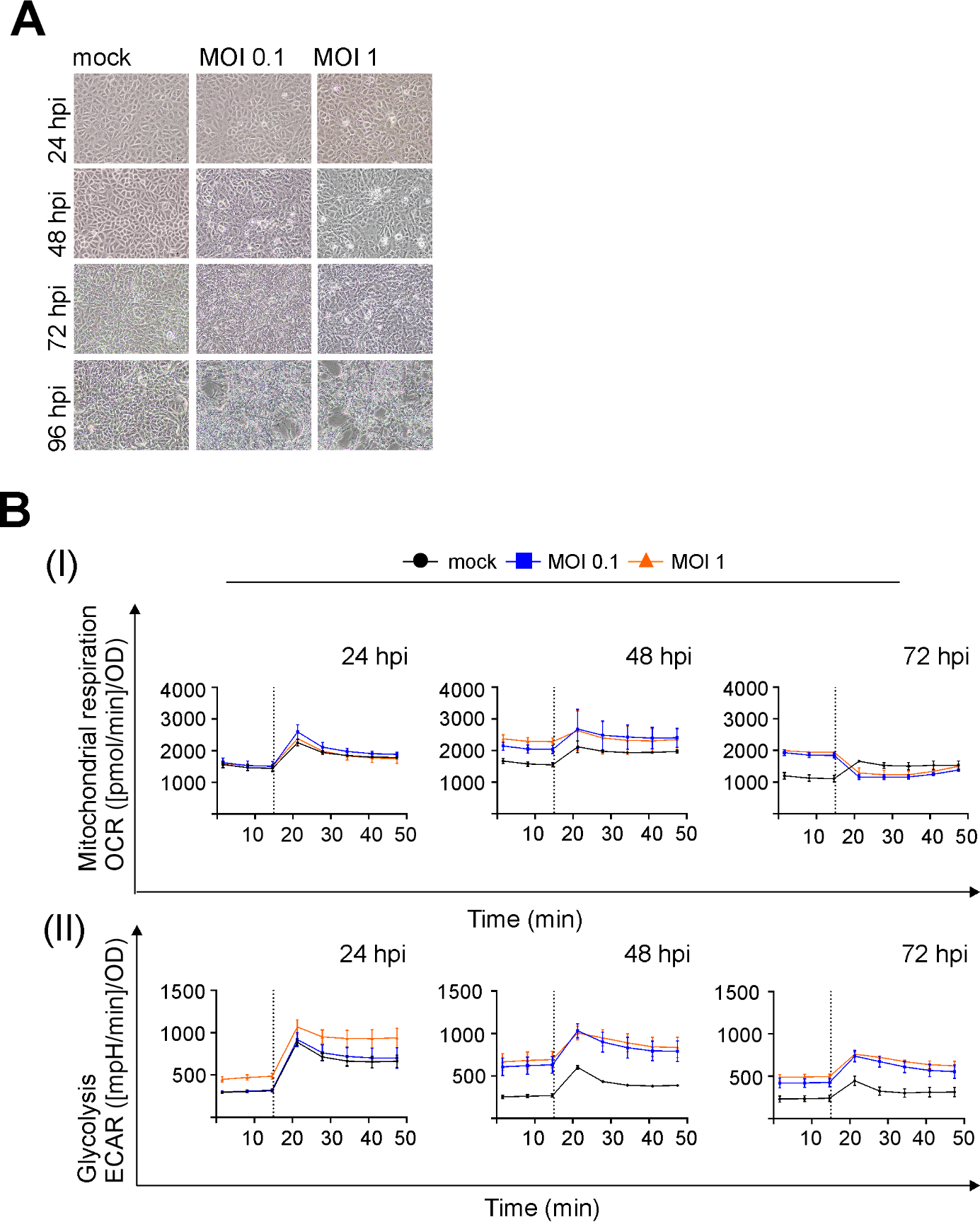
**

**Supplement Figure 1. Cytopathic effect induction on Vero and graphical representation of extracellular flux measurement of USUV- and mock-infected Vero cells at indicated time points. (A)** Phase contrast images of Vero cells over indicated time points for infection with USUV at MOI 0.1 and 1. **(B)** Data in the graphical representation of extracellular flux measurement are shown as mean ± SEM (n=3) of (I) OCR and (II) ECAR over indicated measurement time points as indicative for mitochondrial respiration and glycolysis, respectively. Dashed lines indicate co-injection of 1 µM oligomycin and 0.8 µM FCCP as mitochondrial inhibitors.


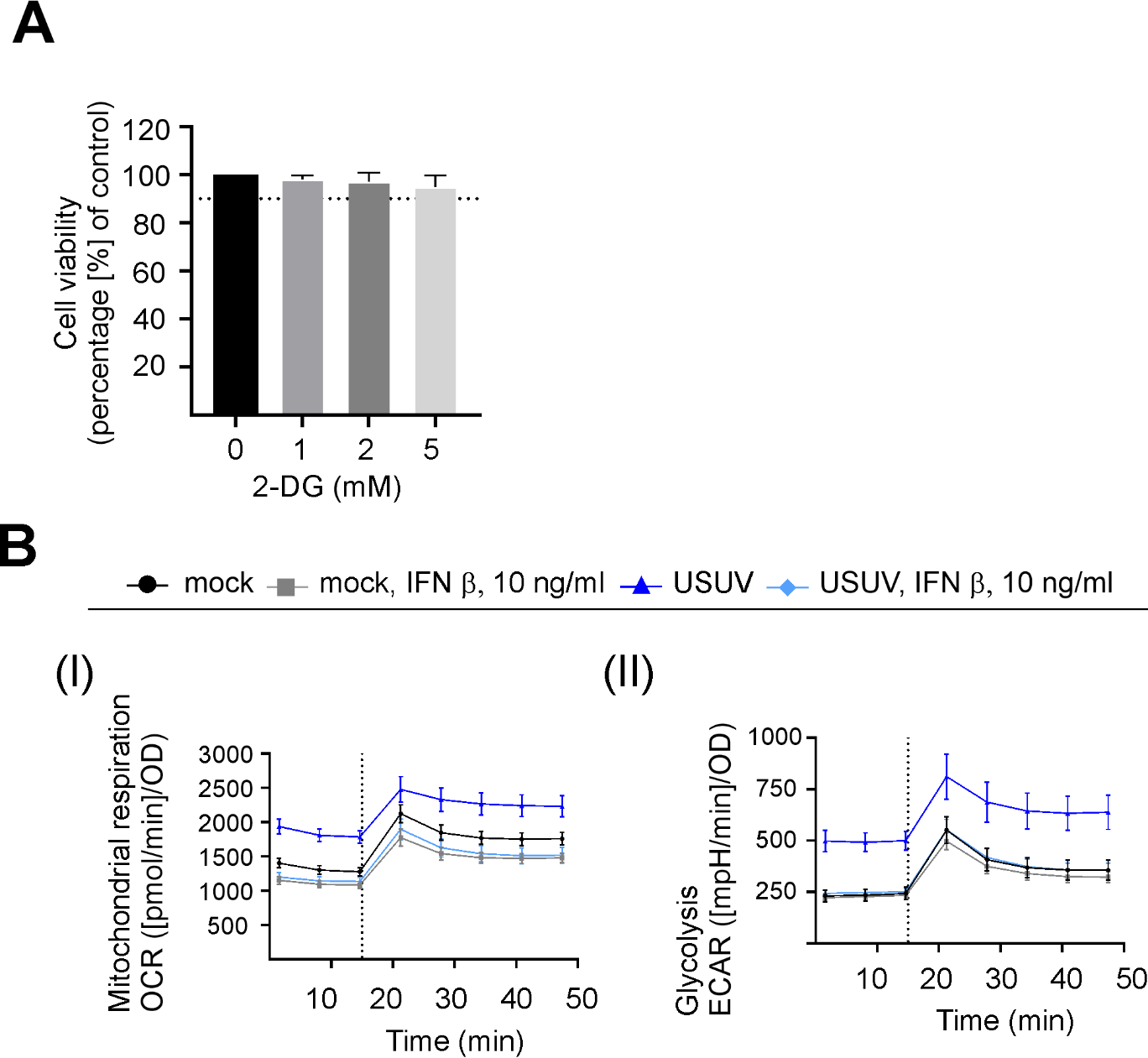


**Supplement Figure 2. Assessment of the potential cytotoxicity of 2-DG and graphical representation of extracellular flux measurement of USUV- and mock-infected Vero cells after addition of IFN β (10 ng/ml). (A)** Cytotoxicity assessment of 2-DG after incubation on Vero cells through the Rotitest assay. Vero cells were exposed to 2-DG at the indicated concentrations for 72 hours. **(B)** Extracellular flux measurement of mock- and USUV-infected Vero cells in the presence of IFN β in comparison to the untreated control. IFN β was added at 24 hpi and extracellular flux measurement was performed at 48 hpi. Data are shown as mean ± SEM (n=3) of (I) OCR and (II) ECAR over indicated time points representative for mitochondrial respiration and glycolysis, respectively. Dashed lines indicate co-injection of 1 µM oligomycin and 0.8 µM FCCP as mitochondrial inhibitors.


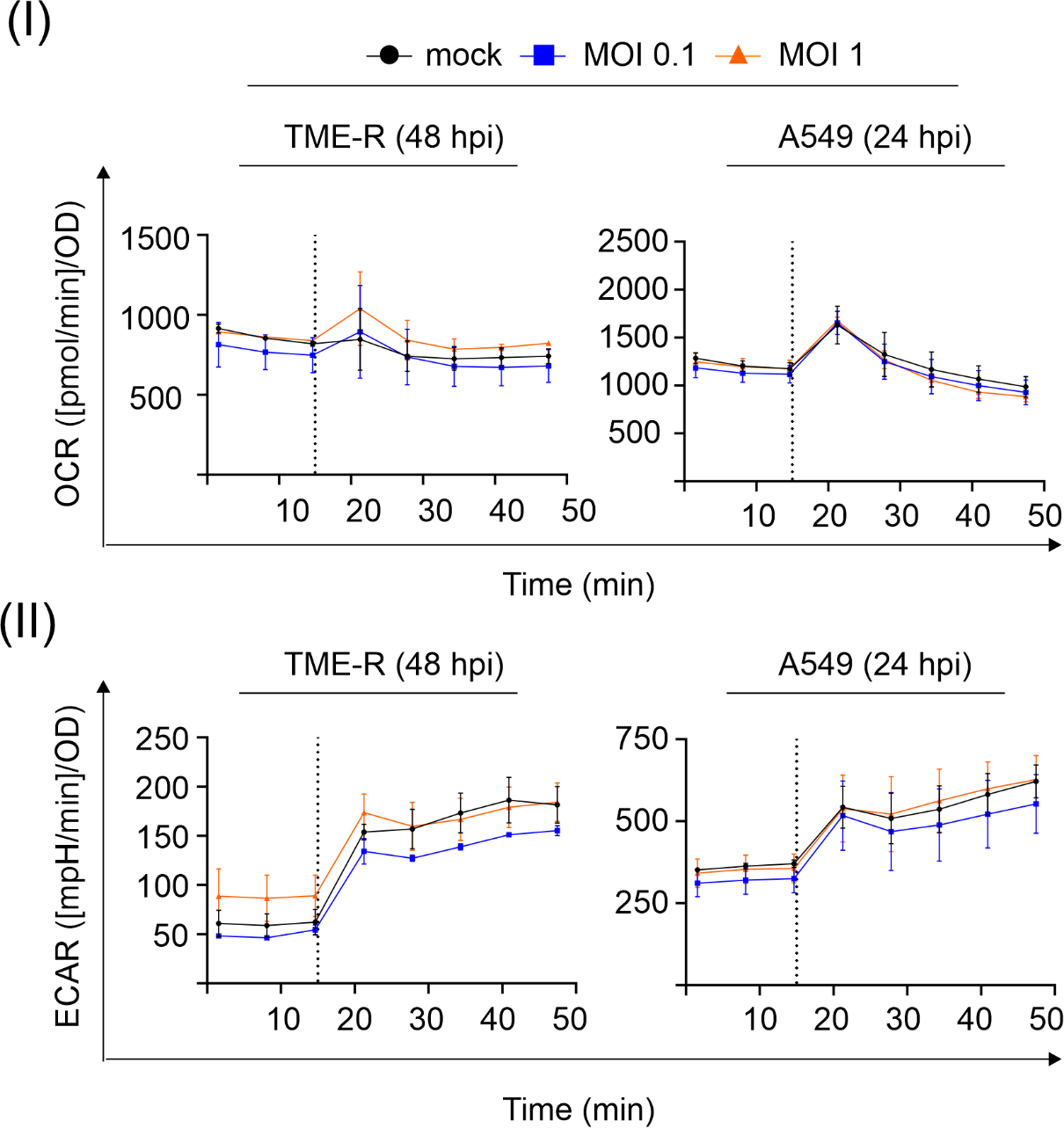


**Supplement Figure 3. Graphical representation of extracellular flux measurement of USUV- and mock-infected avian TME-R and human A549 cells at indicated time points.** Data are shown as mean ± SEM (n=3) of (I) OCR and (II) ECAR over indicated measurement time points as indicative for mitochondrial respiration and glycolysis, respectively. Dashed lines indicate co-injection of 1 µM oligomycin and 0.8 µM FCCP as mitochondrial inhibitors.

**
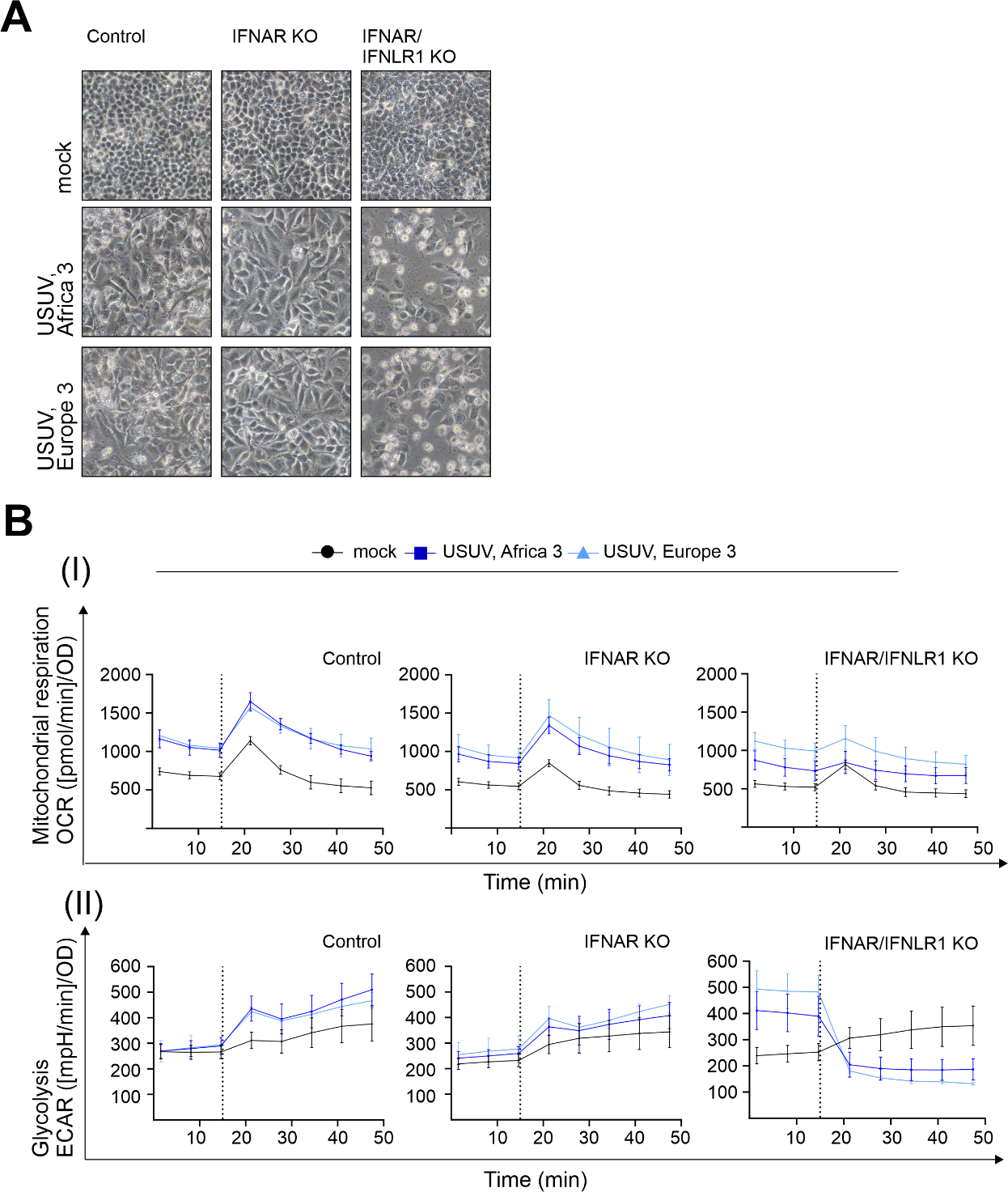
**

**Supplement Figure 4. A549 cells with KO of type I IFN receptor IFNAR and type III IFN receptor IFNLR1 are positive for (A) cytopathic effect development and (B) glycolytic alteration during USUV infection.** **(A)** Phase contrast images of A549 control cells and A549 cells with knockout (KO) of the type I IFN receptor IFNAR either solely or together with the type III IFN receptor IFNLR1 after mock- and USUV-infection. **(B)** Graphical representation of extracellular flux measurement of indicated A549 cells after USUV- and mock-infection. Data are shown as mean ± SEM (n=3) of (I) OCR and (II) ECAR over indicated measurement time point as indicative for mitochondrial respiration and glycolysis, respectively. Dashed lines indicate co‑injection of 1 µM oligomycin and 0.8 µM FCCP as mitochondrial inhibitors.

**
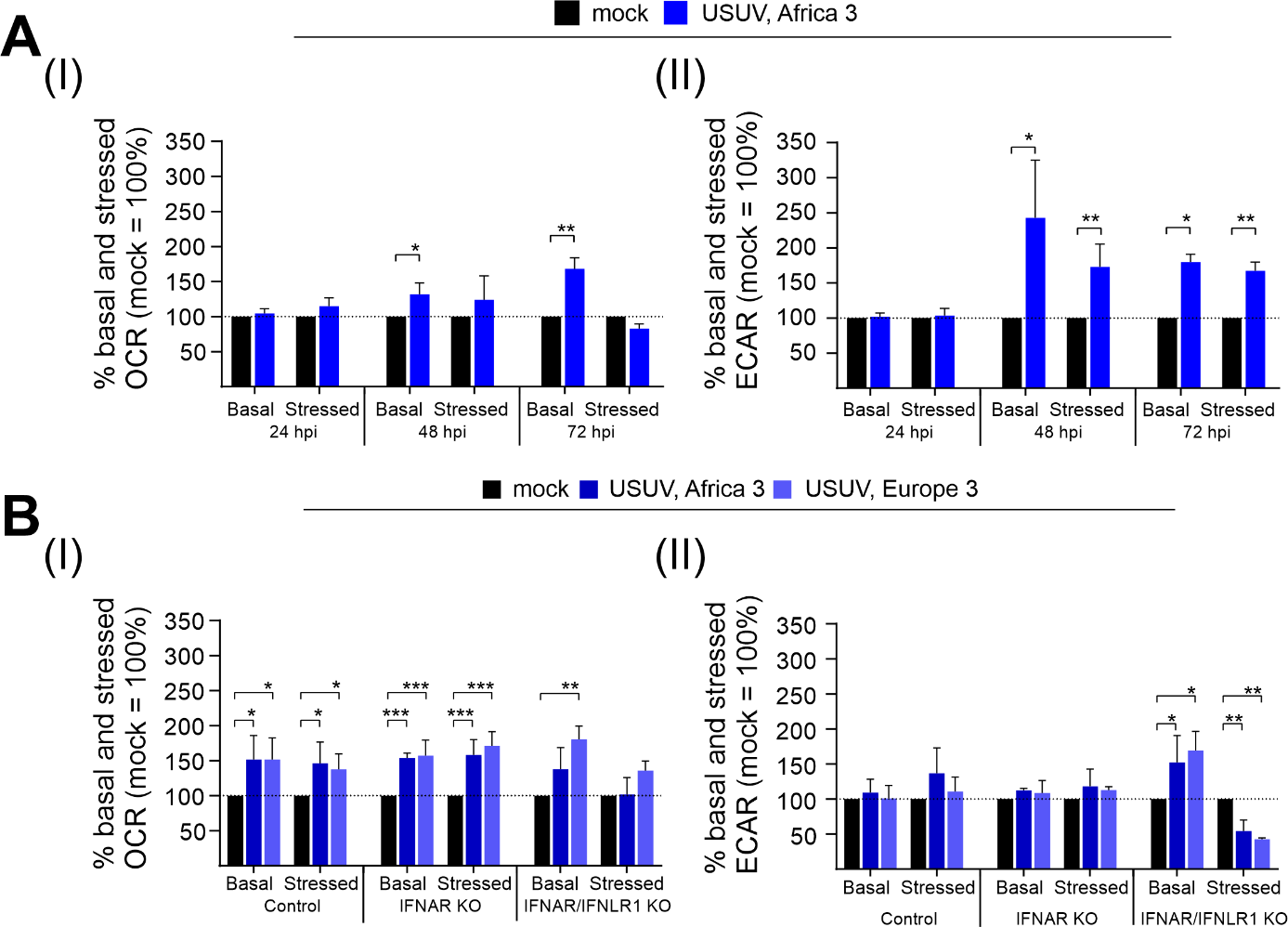
**

**Supplement Figure 5. Assessment of mitochondrial respiration (OCR, [I]) and glycolysis (ECAR, [II]) by extracellular flux analysis in (A) Vero and (B) A549 control and A549 with receptor KO after infection with indicated USUV strains at MOI 0.1.** OCR and ECAR values used for calculation of percent change in metabolic activity after USUV infection in **(A)** and **(B)** are included in Figure 1 and 4, respectively. Data are shown in **(A)** for Vero at indicated time points and **in (B)** for A549 at 96 hpi as percent change in relation to the mock control, which was set at 100%. Statistical analysis was calculated for **log**-transformed data by ANOVA in reference to the mock-infected control. Statistical significance is shown as * p < 0.05, ** p < 0.01 and *** p < 0.001.
